# Malignant solitary fibrous tumor of the pleura

**DOI:** 10.1186/s13019-022-01842-6

**Published:** 2022-05-03

**Authors:** Matthew S. Khouzam, Nayer Khouzam

**Affiliations:** 1 Loyola University Medical Center, Stritch School of Medicine, Maywood, IL 60153 USA; 2 Division of Cardiothoracic Surgery, AdventHealth, Orlando, FL USA

**Keywords:** Solitary fibrous tumor, Dyspnea, Atelectasis, Case report

## Abstract

**Background:**

Solitary fibrous tumors of the pleura are rare diseases of the thoracic cavity. They frequently grow unnoticed until they exert compressive effects on adjacent organs. Treatment of solitary fibrous tumors of the pleura is surgical resection. Post-operative surveillance is recommended to identify early recurrent disease.

**Case presentation:**

We present a rare case of a 76-year-old female patient with no previous pulmonary history who presented with progressive dyspnea, fatigue, and involuntary weight loss. On chest X-ray and computed chest tomography scan, she was found to have a 16.7 cm × 12.8 cm × 10.1 cm bulky mass occupying the left hemithorax with associated compressive atelectasis of the lung. She underwent a computed tomography guided biopsy that revealed the mass to be a solitary fibrous tumor. The patient underwent left muscle sparing lateral thoracotomy with complete resection of the tumor. Post procedure, the left lung fully expanded. 18 months post-resection, she developed a 3.3 cm × 1.7 cm tumor along the left internal thoracic artery lymph node chain which was histologically identical to the resected tumor. The patient is currently being treated with bevacizumab and temozolomide.

**Conclusion:**

Solitary fibrous tumors are very rare pleural tumors. Surgical resection is the treatment of choice followed by close post-operative surveillance.

## Background

Solitary fibrous tumors (SFTs) are rare, mesenchymal neoplasms originating from fibroblastic or myofibroblastic tissue [1]. Although once thought to be a class of mesotheliomas, SFTs are immunohistochemically distinct due to their presence of vimentin, CD34, and lack of cytoplasmic keratins [2–4]. SFTs are most commonly found in the thorax, but have also been identified in extrathoracic locations including the head, neck, breast, abdomen, pelvis, extremities, and scrotum [5, 6]. SFT’s of the pleura (SFTPs) are very rare, occurring at an incidence of 2.8 per 100,000 [7]. They account for < 5% of all pleural tumors, with only approximately one thousand total cases reported in the literature [8, 9]. We present the following case report of a 76-year-old female patient with no previous pulmonary history who presented with dyspnea, fatigue, and anorexia and was found to have a 16.7 cm × 12.8 cm × 10.1 cm SFTP occupying the left hemithorax.

## Case presentation

**Fig. 1:**
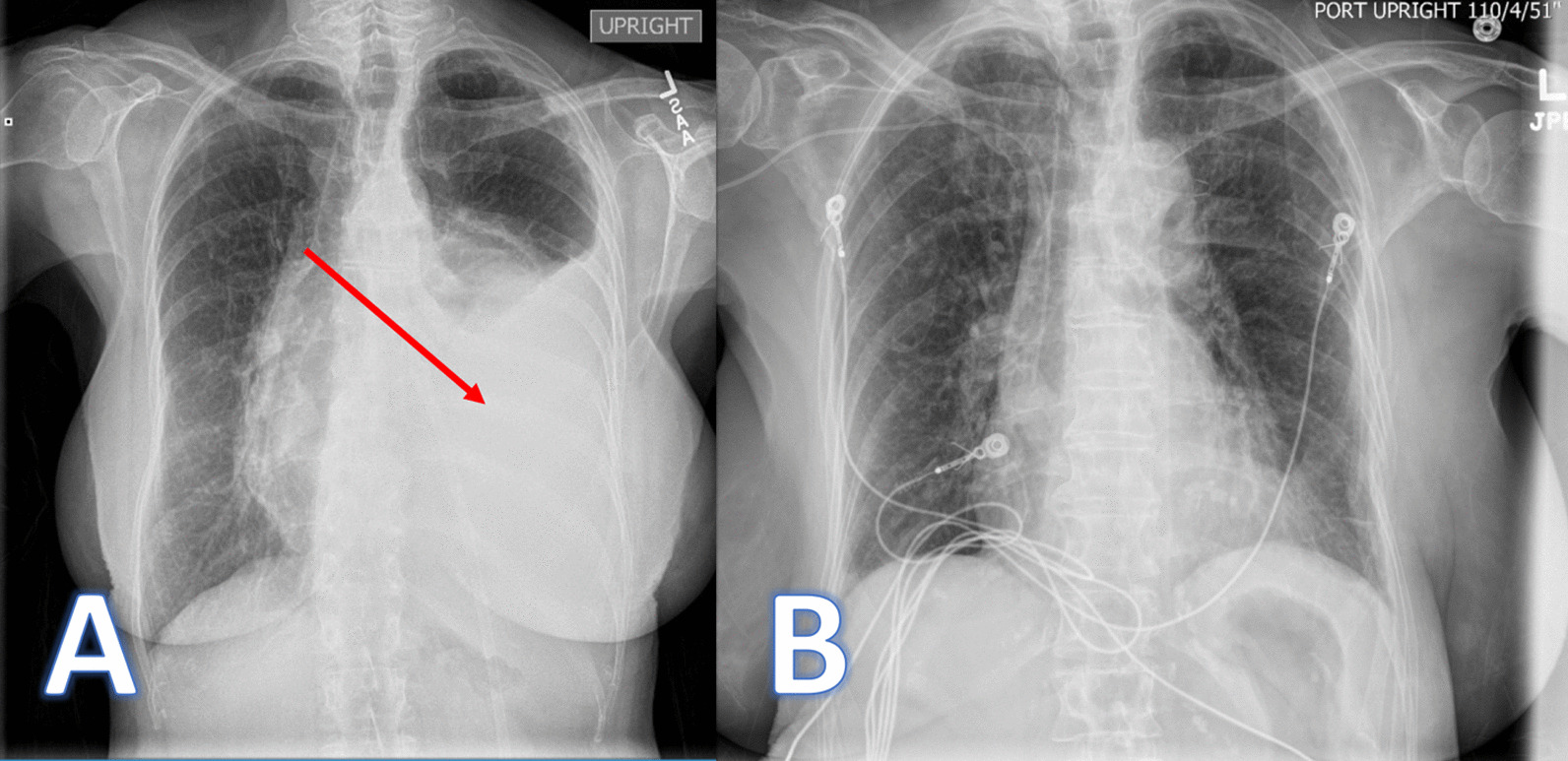
Chest X-rays. **A** Pre-operative chest X-ray showing SFTP. **B** Post-operative chest X-ray

**Fig. 2:**
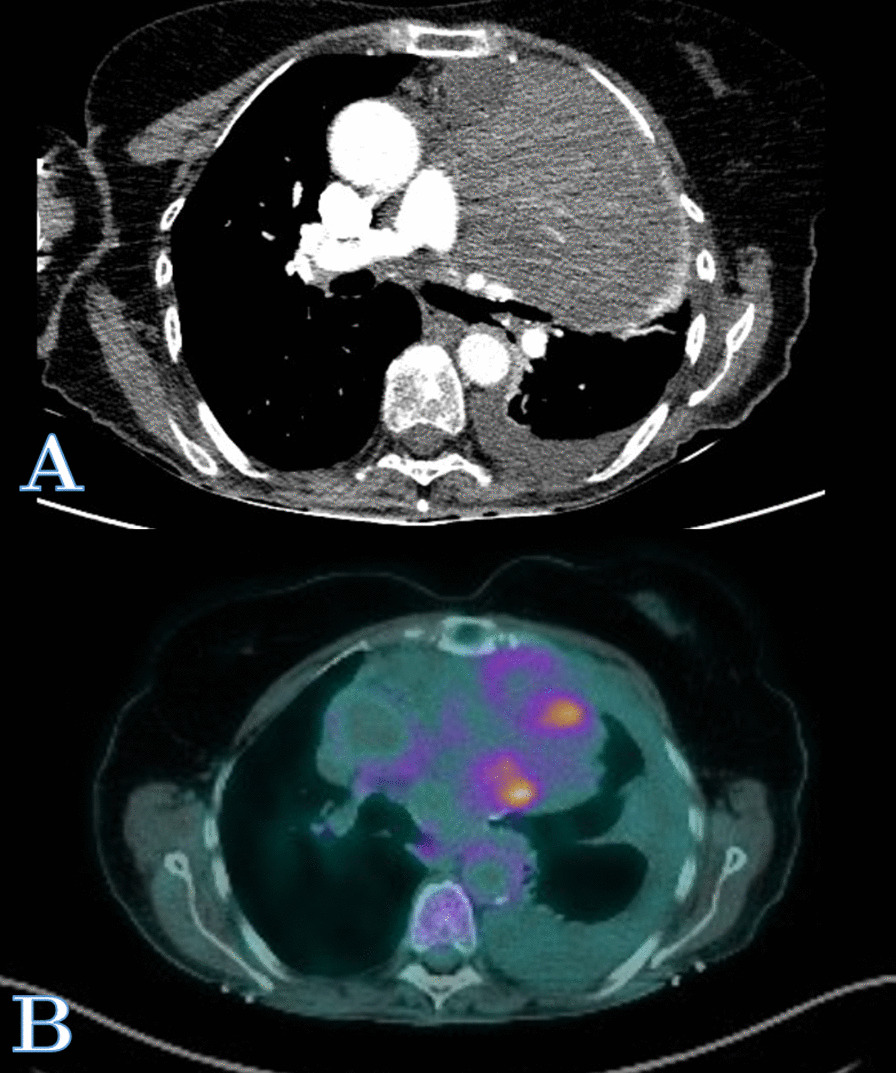
**A** Computed tomography scan with intravenous contrast revealing large bulky tumor with associated left pleural effusion present in the left hemithorax. **B** Positron Emission Tomography scan showing moderate hypermetabolism of the SFTP

A 76-year-old Caucasian female never smoker with no previous pulmonary history was referred to our institution for evaluation of a biopsy proven 17 cm fibrous tumor involving the left hemithorax. The patient related of a two-month history of dyspnea, lack of energy, poor appetite, and 10-pound involuntary weight loss. She denied any fevers, chills, sweats, or hemoptysis. As part of her evaluation, she had a chest X-ray and a computed tomography (CT) scan of the chest that revealed a bulky 16.7 × 12.8 cm × 10.1 cm heterogeneous mass occupying the left hemithorax with a mass effect involving the left side of the anterior mediastinum (Fig. 1). The mass abuts the proximal aortic arch, main pulmonary artery, left pulmonary artery, and left pulmonary vein with mass effect on the pulmonary vasculature. There was no evidence of osseous erosion or abnormal calcification. Also present was a moderate sized left pleural effusion. CT of the chest with intravenous contrast revealed abutment of the tumor to the mediastinum and chest wall without frank invasion (Fig. 2A). Positron emission tomography (PET) scan revealed patchy moderate hypermetabolism with a maximum standardized uptake value (SUV) of 6.7 (Fig. 2B). There were hypoattenuating areas within the mass with central photopenic defect compatible with central necrosis and/or hemorrhage. There was also uptake at the region of her left vocal cord with an SUV of 8.6. She underwent a CT guided core biopsy of the mass with pathology showing a SFT with proliferation of spindle cells in hypo and hyper-cellular areas with a collagenous stroma and foci. By immunochemistry, the spindled cells were positive for CD35, BCL-2, CD99, and negative for S-100, AE1/3, and CAM 5.2. Diagnostic thoracentesis was negative for neoplasia. Her past medical history was significant for craniotomy with resection of a hypoglossal neuroma with resultant left cranial nerve palsy involving cranial nerves 8–12 and tracheotomy. She was evaluated by otolaryngology, and endoscopic examination revealed left vocal cord paralysis which was described as chronic and previous Teflon injection. No tumor was identified.

**Fig. 3:**
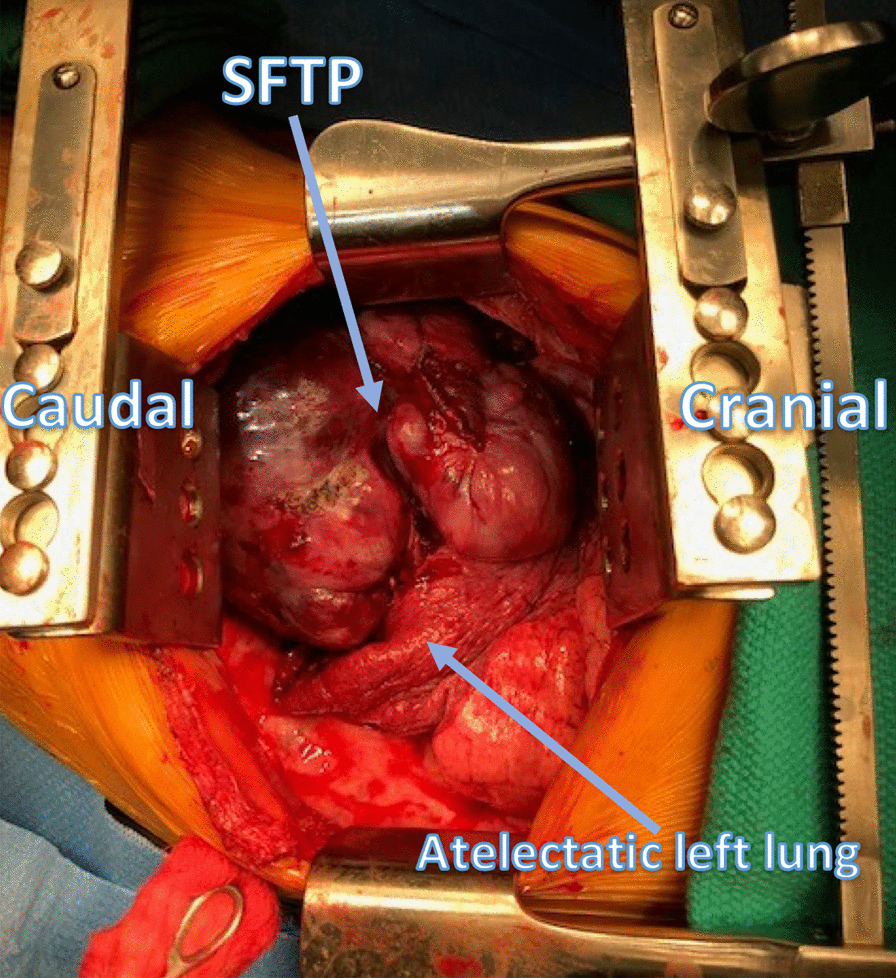
Surgical Image showing SFTP occupying mid and inferior left hemithorax with compressive atelectasis of the lung

**Fig. 4:**
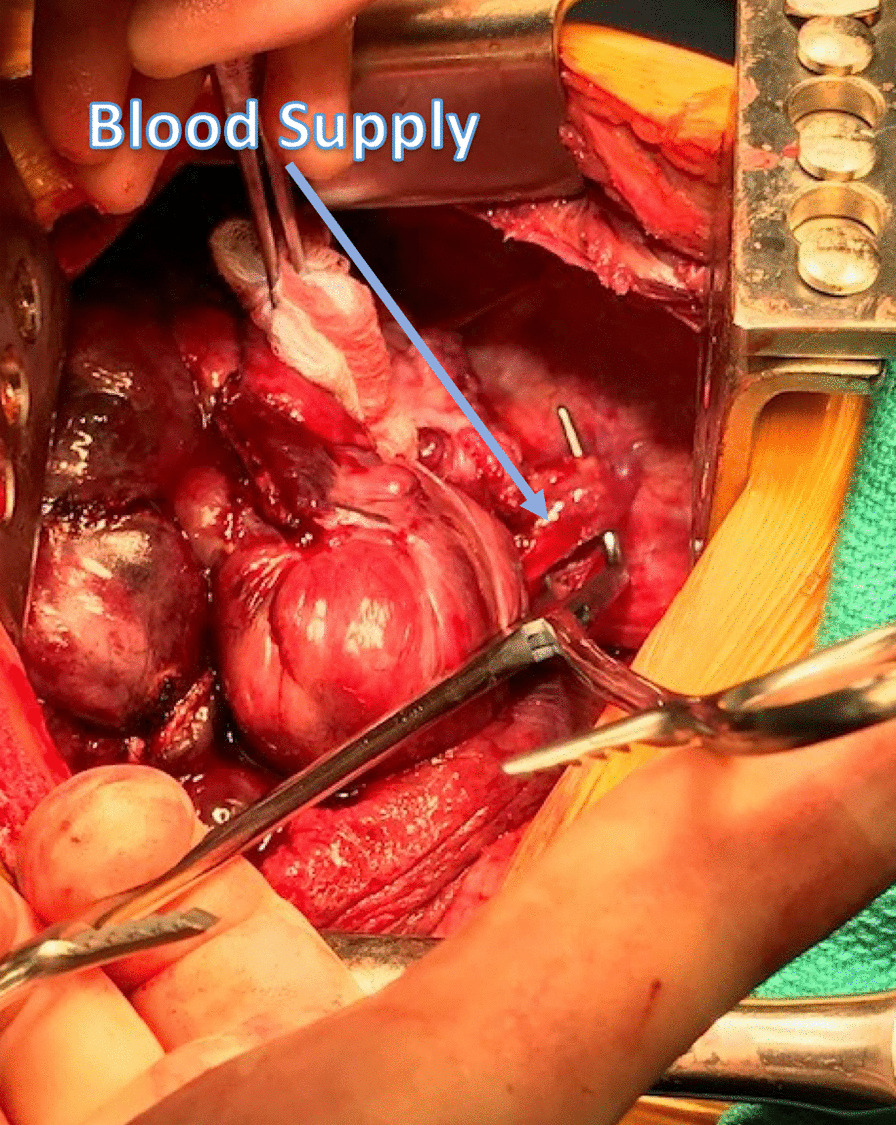
Surgical Image showing blood supply of SFTP from branch of the left internal thoracic artery

**Fig. 5:**
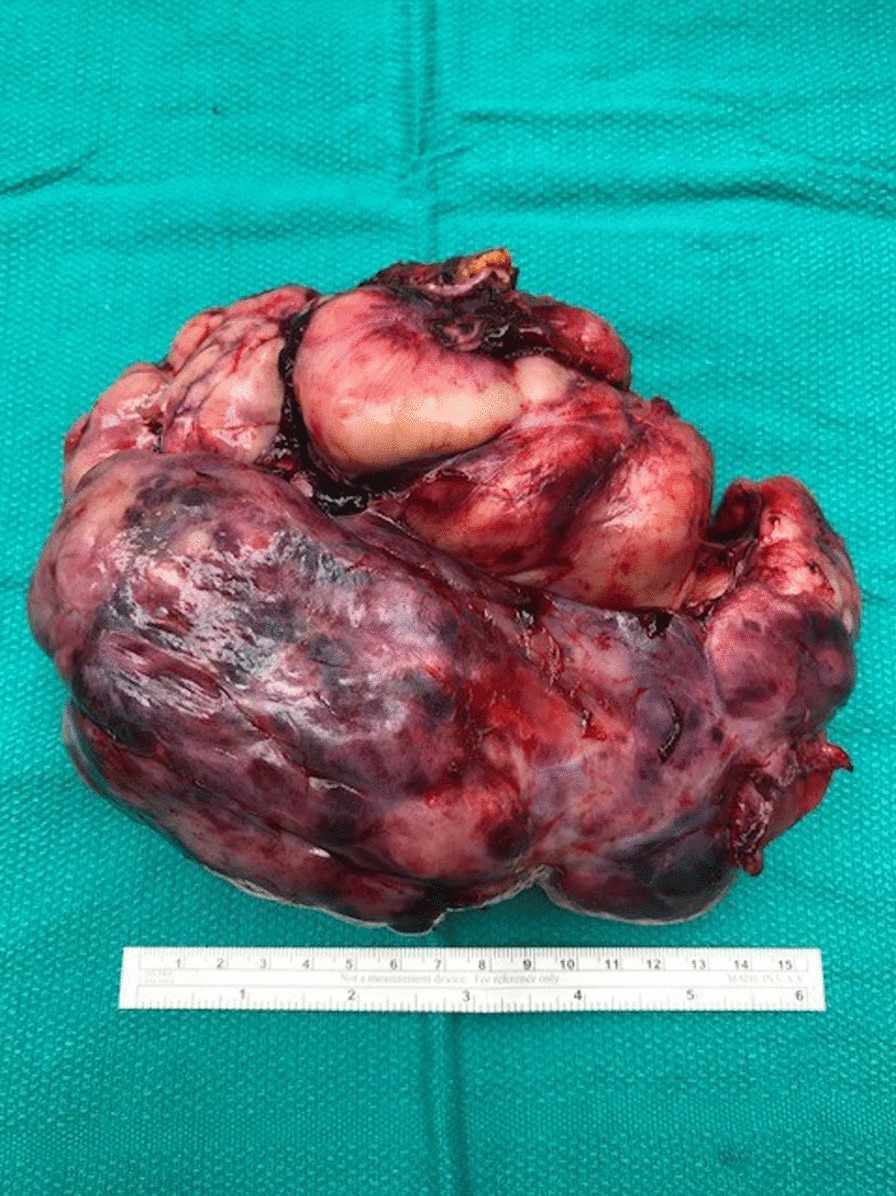
Excised 16.7 cm × 12.8 cm × 10.1 cm SFTP

She was taken to the operative suite. Flexible bronchoscopy revealed no evidence of endobronchial tumor. There was extrinsic compression involving the lingular bronchus and left lower lobe bronchus. She underwent a left muscle sparing lateral thoracotomy. Operative findings revealed a large, bulky, firm, well-circumscribed, vascularized tumor involving the left hemithorax with compressive atelectasis of the left lung (Figs. 3, 4, 5). This was associated with a bloody pleural effusion totaling 1 L. The tumor occupied the anterior and middle mediastinum and was firmly adherent to the left upper lobe lung parenchyma and to the pericardium anterior to the left phrenic nerve. There was no evidence of invasion of the mediastinum. The tumor’s blood supply originated from a branch of the left internal thoracic artery. The patient underwent complete resection of the mass en bloc with portions of the left upper lobe, lingula, and pericardium with ligation of the branch of the left internal thoracic artery (feeding vessel), and thoracic lymphadenectomy. Post resection, the left lung expanded fully.

Cytology of the pleural fluid revealed benign mesothelial cells. Tumor pathology revealed a SFT with atypical features with a mitotic rate of 12/10 high-power field. There was no metastasis to the mediastinal lymph nodes.

There was no lymph or vascular invasion. The tumor was ungraded. 1% of the tumor showed necrosis. All resected margins were free of tumor. No gene mutations were detected in the following genes: KRAS, NRAS. Immunostains of the tumor cells were positive for CD34, CD99, and BCL-2, and were negative for AE1/3, CAM 5.2, S-100 protein and desmin. Her postoperative course was uneventful. She was discharged home on postoperative day five with marked relief of her dyspnea. The case was presented at the multidisciplinary thoracic tumor board. It was recommended that no adjuvant therapy was warranted. The consensus of the board was that the patient should be monitored with a chest CT scan at six-month intervals. 18 months post procedure, she developed a 3.3 cm × 1.7 cm soft tissue tumor along the left internal thoracic artery lymph node chain, a 1.1 cm pre-carinal lymph node, a 1 cm subcarinal lymph node, and a sub-centimeter right and left hilar lymph node. PET scan revealed hypermetabolic uptake in the tumor and lymph nodes with SUV’s ranging between 3.6 and 4.2. CT guided biopsy of the left internal thoracic artery lymph node revealed metastatic fibrous tumor. Tumor markers were identical to that of the resected specimen. She is currently being treated with bevacizumab and temozolomide.

## Discussion

SFTP is a rare mesenchymal spindle cell neoplasm of the thorax. The majority of SFTP originate from the visceral pleura, as was the case with our patient [10]. In extremely rare cases, SFT’s can originate from the lung parenchyma resulting in an intrapulmonary SFT [11]. SFTPs are usually well circumscribed and pedunculated tumors, perfused by vessels within the pedicle [10]. They have a distinct histological fingerprint allowing them to be disambiguated from other neoplasms such as mesothelioma or other lung sarcomas. Differentiation of SFTP includes tissue that is CD34-positive, vimentin-positive, and keratin-negative [2–4]. The majority of SFTP are benign, although when malignant, can paradoxically be CD34-negative [12]. This change in surface protein expression is thought to be due to dedifferentiation of the tumor and often results in poor outcomes [13]. However, this conversion was not found in our patient.

Clinically, SFTPs often present asymptomatically and are often diagnosed as incidental findings in chest X-rays [12]. However, larger SFTPs are usually associated with dyspnea and chest pain [14]. Large tumors can also cause bronchial compression often resulting in atelectasis and very rarely even hemoptysis [15]. The compression of lung tissue along with the tumor mass presents with dullness to percussion upon physical examination [16]. Certain paraneoplastic syndromes such as Doege-Potter syndrome and Pierre-Marie-Bamberg syndrome have been associated with SFTP which can present with digital clubbing, hypertrophic osteoarthropathy, and hypoglycemia due to the production of insulin-like growth factor II from the SFTP [17, 18].

SFTPs can recur even after total surgical resection. However, the likelihood of recurrence is correlated with the tissue type (benign vs malignant) rather than the size of the tumor; benign tumors have an 8% recurrence rate compared to 63% for malignant tumors, even following complete resection [19]. Malignant SFTP, as seen in our patient, are defined as meeting at least one of the following criteria: mitotic rate > 4/10 high-power fields, presence of necrosis, atypical nuclei, and hypercellularity [10]. The majority of SFTPs recur within the first two years of resection. Therefore, follow-up chest radiography or CT scans are recommended at six month intervals in the first two years post-resection [12].

Complete en bloc surgical resection is the primary treatment for SFTP [19]. For small tumors (< 5 cm), thoracoscopic approaches are used for resection, whereas larger tumors often utilize thoracotomy with wedge resection, pneumonectomy, segmentectomy, or lobectomy [20]. Further resections of the chest wall or pericardium may be needed depending on the adhesion borders of the tumor. Post-operative treatment often includes a combination of temozolomide and bevacizumab which are found to have a high disease control when given together [21]. This was given to our patient.

## Conclusion

SFTPs are rare thoracic tumors that can grow silently. Complete surgical resection is recommended for patients with SFTP. Careful post-operative monitoring is advised after surgery due to the possibility of recurrence.

## Data Availability

Not applicable.

## Abbreviations

SFT: Solitary fibrous tumor
SFTP: Solitary fibrous tumor of the pleura
CT: Computed tomography
PET: Positron emission tomography
SUV: Standardized uptake value
